# The Causes of Occupational Accidents and Injuries in Romanian Firms: An Application of the Johansen Cointegration and Granger Causality Test

**DOI:** 10.3390/ijerph18147634

**Published:** 2021-07-18

**Authors:** Larisa Ivascu, Muddassar Sarfraz, Muhammad Mohsin, Sobia Naseem, Ilknur Ozturk

**Affiliations:** 1 Department of Management, Faculty of Management in Production and Transportation, Politehnica University of Timisoara, 300191 Timisoara, Romania; larisa.ivascu@upt.ro; 2 Research Center in Engineering and Management, Politehnica University of Timisoara, 300191 Timisoara, Romania; 3 Binjiang College, Nanjing University of Information Science and Technology, Wuxi 214105, China; 4 School of Business, Hunan University of Humanities, Science and Technology, Loudi 417000, China; mohsinlatifntu@gmail.com; 5 School of Economics and Management, Shijiazhuang Tiedao University, Shijiazhuang 050043, China; Sobiasalamat4@gmail.com; 6 Faculty of Economics and Administrative Sciences, Cag University, Mersin 33800, Turkey; ilknurozturk@cag.edu.tr

**Keywords:** workplace health, accident risk, occupational and fatal accident, safety management, ARDL model

## Abstract

Organizational risks are present in any activity, so it is important to manage them properly. The jobs are dynamic and involve a series of processes and activities. The entire human resource is exposed to several risks. If these risks are approached correctly, the organizational capacity to achieve its objectives and vision will increase considerably. This paper aims to investigate the relationships between work accidents (fatal and non-fatal) and the causes that contribute to their occurrence (causes dependent on the executor, causes dependent on the means of production, workload-dependent causes, and work-dependent causes—the work environment). The augmented Dickey–Fuller (ADF) test is employed to check the data stationarity series, while the Johansen test determines the cointegration relation of variables. The data have been collected from Romanian organizations. The vector error correction model (VECM) and Granger causality test are applied for speed of adjustment, nature, and direction of variables’ relationship. This research demonstrated that both data series are free from the unit-root problem at first difference. The lag length criterions select the third lag for model fitness, and Johansen cointegration declares that variables are cointegrated for the long term. The vector error correction model shows the speed of adjustment from the short to the long run is 83.35% and 42.60% for work and fatal accidents. The study results show that fatal accidents have a series relationship with selected cases for the short run and have a long-run relationship with the means of production. Fatal accidents are directly related to means of production. Fatal accidents are not designed by executors, workload-dependent causes, or work environments in the short run. Fatal accidents are directly related to the means of production and sudden incidents happening in the long run. Fatal accidents are considered by executors, workload-dependent causes, or work environments in the short run. In the long run, fatal accidents are directly related to the means of production and sudden incidents happening.

## 1. Introduction

Improving the competitiveness of the business environment contributes to developing new jobs. The business environment dynamics contribute to the permanent development of new jobs that often require new skills or new work procedures. As well as the European strategy, companies must become involved in organizational approaches, procedures, equipment, and ergonomics to provide sustainable jobs for the company’s employees. To improve these jobs, work accidents and the factors contributing to their occurrence must be investigated. Carrying out an inventory of work accidents, an increase in their number is observed [1]. At the level of the European Union, in 2010, there were 153,461,300 employees, and in 2019, the number of employees increased by approximately 9%. As a result, the number of employees reached 166,999,300 in 2019 [2]. Following current statistics, it is observed that an increase in the number of employees contributes to the increase in the number of accidents.

From the perspective of the number of accidents, in 2010, their number was 2,657,234, and in 2019, there were 3,124,828 accidents. Among the injured employees, 2,137,935 are men, and 986,107 are women. The number of fatal accidents is 3332 accidents [2]. The accident rate in the EU shows a value of two fatal accidents per 100,000 employees in 2019. The sectors that are most affected by fatal accidents are construction (24%), manufacturing (19%), and logistics (19%). There is a 15% increase in the number of accidents in 2019 compared to the reference year, 2018 [2].

In 2010, there were several 4,376,044 employees in Romania. This number has increased annually, reaching a level of 5,164,471 employees in 2019. Evaluating these data, we can see an increase in the number of employees by about 15% in 2019 compared to 2010. These new jobs involve responsibility, building working conditions, procedures, and relationships between employees. These jobs developed 3622 work accidents in 2010. In 2019, there were 5145 work accidents, 20% more than in 2010. It can be seen that in 2019, the number of employees increased, but also the number of work accidents. Even the percentage of accidents is higher than the percentage increase in the number of employees. It can be concluded that new jobs can be more complex and lead to more accidents (National Institute of Statistics, 2020). It is not easy to access injuries data caused by risky materials [3]; so, developing preventable measure strategies is difficult [4].

This paper investigates occupational accidents, considering the causes of their occurrence. The causes considered in this research are causes dependent on the executor, causes dependent on the means of production, workload-dependent causes, and work-dependent causes—the work environment. The present research begins to present the specialized terms, the existing research situation at the global level, and the factors contributing to occupational risks. The augmented Dickey–Fuller (ADF), Johansen cointegration test, vector error correction model (VECM), and Granger causality test are employed for the first time to determine the relationship of work and fatal accidents with divergent cases. According to the legislation in force, a work accident is defined as “violent injury to the body, as well as acute occupational intoxication, which occur during the work process or in the performance of duties and which cause temporary incapacity for work of at least three calendar days, disability or death.” The work accident leads to the death or injury of the employee. This accident occurs during the work process or work duties, the disappearance of a person or traffic accident that occurs on the employee’s route from home to work, a dangerous incident, and occupational diseases or related professions [5].

The specialized literature presents a series of studies regarding work accidents. Some of them only target specific areas of work, for example, construction [6,7], transport [8,9,10,11,12], industry [13,14,15,16,17], and other domains. Among the causes of construction, accidents are deficiencies related to the management of the work team (over 75% of accidents), problems related to the workplace (over 50%), deficiencies with the equipment and work procedures (56%), problems related to the materials used (35%), and deficiencies of the working environment [18,19,20]. Among the causes of work accidents in the transport sector are: short sleep intervals (less than 4 h of sleep in an analyzed period) and causes dependent on equipment and infrastructure [8,21,22]. Non-fatal accidents have been intensifying in recent days [23,24].

In industry, accidents occur because of causes dependent on equipment, the work environment, or the worker. Accidents occur mainly by external employees. Many accidents are registered for outsourced operations [25,26,27]. That is why it aims to train and plan work tasks to reduce the accident rate in the industry [8,9,10,11,12,28]. Accidents at work in recent years have increased, with various causes contributing to increased absenteeism in organizations [29,30]. It is vital to make an inventory of work accidents and contribute to their development because the dynamics and current complex working conditions can develop more accidents. It can be seen that both in the EU and Romania, there is an increasing trend regarding workplaces and work accidents. It can be said that an increase in the number of jobs leads to an increase in the number of accidents.

Accidents at work in different fields of activity fall in specific directions. These directions concern the worker, the work environment, the production process, or the employee [31,32]. Depending on the severity, the two categories for work accidents can be delimited: non-fatal and fatal accidents at work [30,33]. Therefore, these work accidents are caused by causal elements. The systemic principle of approaching the work processes requires expressing the causes on the four elements: the executor, the means of production, the workload, and the work environment. This procedure is also followed if the work process is complex or less complex. The work process elements are characterized by attributes, states, phenomena, and behaviors. These features can lead to a system malfunction. This dysfunction can cause accidents at work and/or occupational diseases, together called risk factors. These risk factors can be potential causes of accidents at work or occupational diseases. If an accident at work or occupational disease has occurred, the causes of these elements must be identified. The government should increase work accident data quality [34].

The source of the data series is the Romanian Ministry of Labor and the Romanian National Institute of Statistics. These data series show the total number of accidents at work (fatal and non-fatal). Therefore, the fields of activity are considered as evaluated by the Ministry of Labor: retail trade, land transport, building construction, wholesale trade; manufacture of road transport vehicles, trailers, and semi-trailers; the food industry; human health activities; industry of metal constructions and metal products, exclusive machines, equipment, and installations; production and supply of electricity and heat, gas, hot water, and air conditioning; manufacture of furniture; public administration; wood processing, and other activities.

The paper presents an analysis of accidents in Romania for the period 2010–2019. At the Romanian level, it is necessary to analyze the number of accidents and a clear presentation of their causes because the existing research is restrictive. The present study advances the research carried out so far by the methodology used and by the long period of analysis (10 years). Thus, an inventory of the current situation is developed by presenting some necessary hypotheses. This study is of interest to researchers, authorities, practitioners, and companies to develop strategies to reduce the likelihood of risks.

## 2. Materials and Methods

The causes of work accidents are divided into four categories: causes dependent on the executor—the injured (C1), causes dependent on the means of production (C2), workload-dependent causes (C3), and work-dependent causes—the work environment (C4). Cause 1 refers to falls, omissions, incorrect operations, non-use of protective equipment, or other dangerous conditions. The working conditions that constitute C1 can significantly impact the number of work accidents. The causes dependent on the means of production are relevant for work accidents. Often, production equipment is not adequately balanced, its handling conditions can cause minor or severe accidents, and working positions for their use can contribute to accidents of varying severity. The workload, C3, can be as significant as the first two conditions. Deficiencies in orientation, omissions in work operations, and other conditions are essential for reducing work accidents and assessing significance. Work-related causes, C4, are critical occupational hazards. The workplace climate or the environment’s characters are essential conditions that must be managed to correct the occupational risks. The causes of fatal accidents are the four leading causes of the present study: causes dependent on the executor—the injured (C1), causes dependent on the means of production (C2), workload-dependent causes (C3), and work-dependent causes—the work environment (C4). From the C1 perspective, falls, omissions, and some operations can contribute to fatal accidents. Additionally, not using protective equipment can cause fatal accidents. The causes dependent on the means of production are the leading causes of fatal accidents. In some situations, workload, C3, can be fatal to the employee. This high workload can overwhelm the employee and ultimately lead to death. If significant omission occurs, it can be fatal to employees. That is why this primary cause, C3, is considered significant for fatal accidents. The workplace climate or the nature of the environment that are conditions for the main cause of C4 can be the cause of fatal accidents. All the main causes’ conditions must be evaluated through efficient risk management.

To reduce the severity of the causes of work accidents or occupational diseases, the elements should be identified at each department’s level to specify as clearly as possible the place of action and prevention measures and the employer’s responsibility in case of an accident. Following Law 319/2006, the causal elements are divided into four categories. The employer must declare the occurrence of an accident and must identify all the causal elements and working conditions. These data are transmitted through a nationally agreed report. The causal elements that contribute to the occurrence of work accidents are:

- Causes dependent on the executor—the injured.
- Causes dependent on the means of production.
- Workload-dependent causes.
- Work-dependent causes—the work environment

A work accident can have one or more causal elements. The State Committee for Labor Protection establishes the classification of cases. Causes dependent on the executor—the injured—involve the improper performance of process operations. As a result, dangerous and harmful conditions develop. Causes depending on production refer to the machines’ functional movements. Mechanisms triggered contraindicated incompletely described procedures for functional movements and other activities depending on production. Workload-dependent causes refer to the improper distribution of executors in the workplace. In some companies, no training is provided in occupational safety and health, or this training is provided at a low level. Work-dependent causes—the work environment—include the work climate. Depending on the field of activity, the work environment may have different particularities.

### 2.1. Data Description

This research is based on quarterly data of work and fatal accidents (mentioned the specification of firms/companies/road accidents) from 1 January 2010 to 31 December 2019. The data used in this research were collected from the Romanian Ministry of Labor and the National Institute of Statistics. The employer immediately reports an event that has contributed to the body’s death or injury to the Territorial Labor Inspectorate.

**Figure 1:**
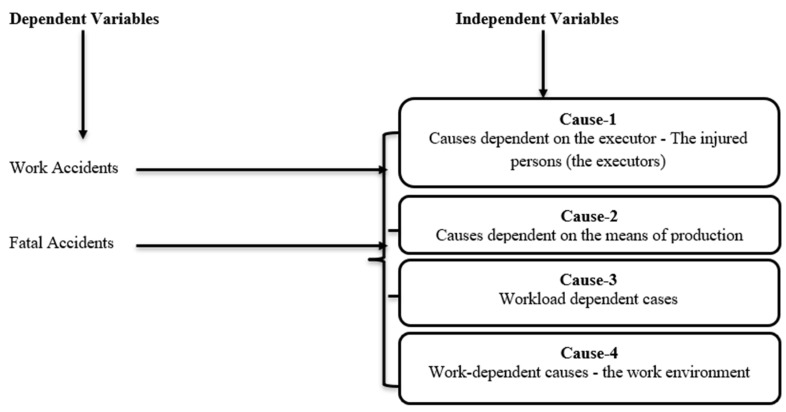
Conceptual framework.

The work and fatal accidents are considered dependent variables, while four main causes of work conditions are declared independent (see Figure 1). The main causes cover different work conditions, which are explained in detail in Table 1. The main causes are computed by the weighted average method for analysis.

The number of accidents considered in the present research is presented in the following table. Each accident had one or more causal elements. These are accidents reported by employers.

### 2.2. Research Methods

**Figure 2:**
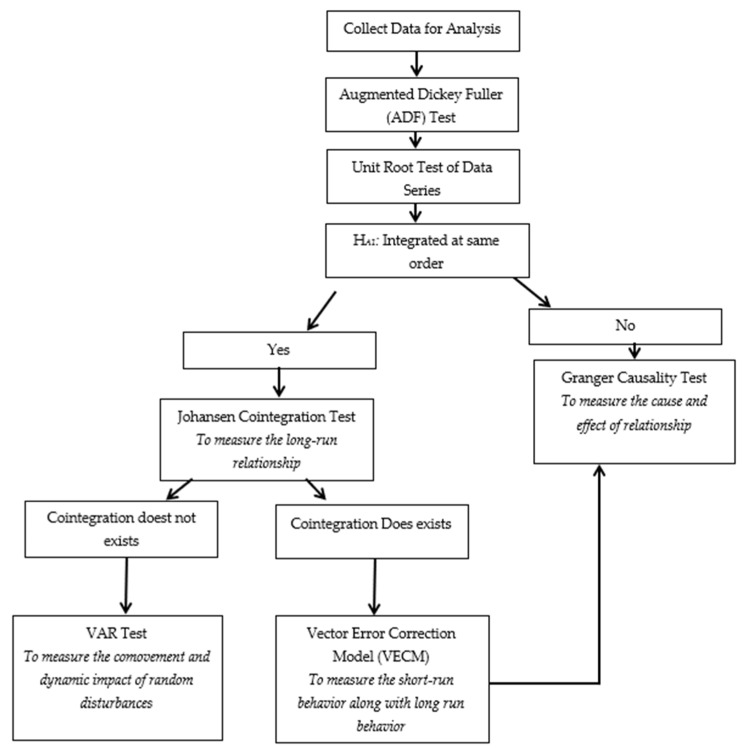
Flowchart of methodology.

The research methodology for this article is alienated into four sub-sections that are (1) stationarity test or unit-root test. (2) Cointegration test is employed after confirmation of integration order and stationarity of data series. In this research, the Johansen cointegration test is employed to check long-run relationships or long-run equilibrium among series with a linear combination of variables. (3) The vector error correction model (VECM) is used to determine the causality direction of variables after confirmation of the cointegration relation. The fourth method is the Granger causality test used to check individual variable direction and relationships with another individual. A flowchart of the complete methodology is pictorially presented in Figure 2. In this study, Eviews-10 is used to run the Johansen cointegration model, VECM, and Granger causality test for data analysis.

#### 2.2.1. Unit-Root Test

The unit-root test is used to verify the order of variables’ integration. As per existing literature, the augmented Dickey–Fuller (ADF) is a well-known test for checking the integration order. This research is also employed the ADF test for unit root by the following equation:

(1) $$\Delta Y_{t}=\alpha +\beta_{t}+\rho Y_{t-1}+\overset{k}{\underset{i=1}{\sum }}\gamma_{i}\Delta Y_{t-1}+e_{t}$$

where $\Delta Y_{t}=Y_{t}-Y_{t-1}$; ${\Delta Y}_{t-1}=Y_{t-1}-Y_{t-2}$; Δ is difference operator; α is constant, β is coefficient on-time trend t, ρ represents that the number of lags is empirically determined using Schwarz information criteria (SIC), and $e_{t}$ is an error term with zero mean and variance. The coefficient term $Y_{t-1}$ is included later for testing the significance of coefficient [35,36,37]. The augmenting process is completed with the possible removal of autocorrelation among error terms. If:

(2) $$Calculated\;Value>Critical\;Value=Reject\;H_{0}\;and\;H_{0}=A\;unit-root\;is\;present\;in\;\;Y_{t}\;.$$

(3) $$Calculated\;Value>Critical\;Value=Accept\;H_{1}and\;H_{1}=\;Y_{t}\;has\;stationarity\;.$$

#### 2.2.2. Cointegration Test

After checking the order of integration and stationarity of series, the second stage of analysis is tested. The cointegration test measures long-run relationships or long-run equilibrium among many time series datasets with a linear combination of variables. This research employs the Johansen cointegration test to check the stability and long-term equilibrium relationship between variables by the following equation:

(4) $$\Delta Y_{t}=\Pi_{t-1}+\overset{p-1}{\underset{i=1}{\sum }}\Gamma_{i}\Delta Y_{t-1}+Bx_{t}+\mu_{t}$$

(5) $$\Pi =\overset{p}{\underset{i=1}{\sum }}A_{i}-I,\Gamma_{i}=-\overset{p}{\underset{i=t+1}{\sum }}A_{j}\;$$

$H_{0}=The\;Cointegration\;exit\;in\;series.$

$H_{1}=The\;Integration\;exist\;in\;series.$

The above equation shows Π is an indicator of the adjusted disequilibrium matrix. The stacking coefficient A is boosted up endogenous factor’s speed of change counter to disequilibrium. The sign Γ is used to capture the short-term dynamic adjustment [37,38,39]. This test progression can declare the association of variables with their positions in the matrix and featuring roots.

#### 2.2.3. Vector Error Correction Model

The vector error correction model (VECM) is used to determine the causality direction of variables after confirmation of cointegration relation [40,41]. The VECM framework is structured as follows:

(6) $$\left[\begin{array}{c} \Delta lnCause-1_{t}\\ \Delta lnCause-2_{t}\\ \Delta lnCause-3_{t}\\ \Delta lnCause-4_{t} \end{array}\right]=\left[\begin{array}{c} \theta_{1}\\ \theta_{2}\\ \theta_{3}\\ \theta_{4} \end{array}\right]+\left[\begin{array}{c} d_{11m}d_{12m}d_{13m}d_{14m}\\ d_{21m}d_{22m}d_{23m}d_{24m}\\ d_{31m}d_{32m}d_{33m}d_{34m}\\ d_{41m}d_{42m}d_{43m}d_{44m} \end{array}\right]\times \left[\begin{array}{c} \Delta lnCause-1_{t-1}\\ \Delta lnCause-2_{t-1}\\ \Delta lnCause-3_{t-1}\\ \Delta lnCause-4_{t-1} \end{array}\right]+\ldots +$$

(7) $$\left[\begin{array}{c} d_{11n}d_{12n}d_{13n}d_{14n}\\ d_{21n}d_{22n}d_{23n}d_{24n}\\ d_{31n}d_{32n}d_{33n}d_{34n}\\ d_{41n}d_{42n}d_{43n}d_{44n} \end{array}\right]\times \left[\begin{array}{c} \Delta lnCause-1_{t-1}\\ \Delta lnCause-2_{t-1}\\ \Delta lnCause-3_{t-1}\\ \Delta lnCause-4_{t-1} \end{array}\right]+\left[\begin{array}{c} \lambda_{1}\\ \lambda_{2}\\ \lambda_{3}\\ \lambda_{4} \end{array}\right]\left(ECM_{t-1}\right)+\left[\begin{array}{c} \varepsilon_{1t}\\ \varepsilon_{2t}\\ \varepsilon_{3t}\\ \varepsilon_{4t} \end{array}\right]$$

where the coefficients $\lambda_{1}-\lambda_{7}$ are indicated the error correction term, the homoscedastic disturbance term is denoted by $\varepsilon_{1t}-\varepsilon_{4t}$, and $ECM_{t-1}$ represents long-run equilibrium and speed of adjustment.

#### 2.2.4. Granger Causality Test

The Granger causality test estimated by the following equation:

(8) $$X_{t}=\alpha_{0}+\overset{k}{\underset{j=1}{\sum }}\alpha_{1s}X_{t-s}+\overset{m}{\underset{i=1}{\sum }}\alpha_{2i}Y_{t-m}+\varepsilon_{1t}$$

(9) $$Y_{t}=\beta_{0}+\overset{n}{\underset{j=1}{\sum }}\beta_{1j}Y_{t-j}+\overset{p}{\underset{h=1}{\sum }}\beta_{2h}X_{t-h}+\varepsilon_{2t}$$

In the above equation, it is assumed that the term $\varepsilon_{1t}$ and $\varepsilon_{2t}$ are uncorrelated with each other as $E\left(\varepsilon_{1t},\;\varepsilon_{2t}\right)=0=E\left(\varepsilon_{2t}\varepsilon_{2s}\right)\ldots ..s\neq t$. The unidirectional causality from fatal and work accidents to 4 specific causes is shown in the equation. If estimated coefficient $\alpha_{2i\;}$ is statistically significant, $\;\alpha_{2i}\neq 0\;then\;Y\to Granger\;causes\to X$ [42]. If *X* is cause variable for *Y* and $\beta_{2h}$ is statistically significant, i.e., $\beta_{2h}\neq 0$ [43,44]. The significance of $\;\alpha_{2i}$ and $\beta_{2h}$ confirms mutual dependency of two specific variables. The term *Y* and *X* will be independent if $\;\alpha_{2i}$ and $\beta_{2h}$ are not other than zero.

## 3. Results

In Table 2, augmented Dickey–Fuller (ADF) results for integrating work and fatal accidents with four main causes are presented. The null hypothesis of a unit root in series has failed to reject at a level even at a 10% level of significance [45]. Both dependent variables series, i.e., work and fatal accident, also obtained stationarity at the first difference at 1%. The stationarity of the series under work and the fatal accident is confirmed after the first difference. After taking the first differences, both conditional series have shown 1% significance level for all causes except C-2 of a fatal accident. The C-2 of the fatal accident of ADF is significant at a 5% level of confidence. The last column contains the order of integration, which is representative of the integrated variables order [46,47]. The numeric values 0 and 1 are used for level and first difference. The below table showed I (1) in front of all causes because the results support the first difference’s significance, which is a fundamental requirement to run the cointegration test.

Table 3 shows the results of a second step in which an appropriate lag length is selected. Based on VAR lag order selection criteria, lag three is chosen for work accidents by three information criteria, i.e., Akaike information criterion (AIC), Schwarz information criterion (SC), and the Hannan–Quinn information criterion (HQ), while in fatal accidents, AIC and HQ chose lag three and SC chose lag 0 as an appropriate lag.

After selecting the appropriate lag length, the next step is to check long-run relationships among variables used in research. The Johansen cointegration test is employed to check the long-run relationship among variables and is presented in Table 4. The results are in two portions, i.e., the Johansen trace test and max eigenvalue. The results indicated that variables are cointegrated with none 83.83758 for trace (work accident), 76.77265 for trace (fatal accident) at a 1% level of significance. The value of the maximum eigenvalue for work accidents is 41.83049, and the value for a fatal accident is 36.07142, which are significant at 1% and 5% levels, respectively. The cointegration results confirmed the long-run relationship between work and fatal accidents with four leading causes.

In the next step, the trace and max eigenvalues test results generated a cointegration equation based on log-likelihood (LL) ratio. A linear combination between selected causes with work accident and the fatal accident could be scrutinized from the cointegration equation. This cointegration equation is also used to check long-run relationships, and it is confirmed for this research. In Table 5, The normalized work accident and fatal accident equations showed a mixed relationship—the significant positive relationship for C-3 and C-4 and a negative relationship for C-2 with work accidents. Simultaneously, the fatal accident is positively related to C-2 and C-3 and has a significant relationship with C-4.

In Table 6, the vector error correction model (VECM)’s results are presented with multiple time series’ long-run and short-run relationship between a dependent (work and fatal accidents) and independent variable (four main causes). The short-run causality test shows unidirectional causality between work accident and cause-3 with a first difference $\left(WA_{-1}=>C3_{-1}\neq WA_{-1}\right)$ at a 10% level of significance. A unidirectional causality confirmed between cause-1 and cause-3 $\left(C1_{-1,-2,-3}=>C3_{-1,-2,-3}\neq C1_{-1,-2,-3}\right)$ with three different structures at a 1% level. The cause-1 unidirectional related to cause-4 $\left(C1_{-1}=>C4_{-1}\neq C1_{-1}\right)$ at a 10% level of confidence. The unidirectional causal effect of cause-1 and cause-3 on work accidents $\left(C2_{-1}=>WA_{-1}\neq C2_{-1}\right)$ $\left(C4_{-1,-2,-3}=>C3_{-1,-2,-3}\neq C4_{-1,-2,-3}\right)$ $\left(C1_{-3}=>WA_{-3}\neq C1_{-3}\right)$ with different subscripts is confirmed at a 1% level of significance. The error correction term (ECT_t-1_) meets the requirement of negativity and significance at a 1% level, supporting the relationship between dependent and independent variables. The ECT value declares the 83.35% adjustment speed of short-run causality into the long run. The ECT of cause-1, 2, and 4 is positively significant, while cause-3 is negatively insignificant. The unidirectional relationship is observed between fatal accident and cause-2 with one difference $\left(FA_{-1}=>C2_{-1}\neq FA_{-1}\right)$ at 1% and cause-2 with a difference of three $\left(C2_{-3}=>FA_{-3}\neq C2_{-3}\right)$ significant at a 5% level of significance. A bidirectional relationship observed between fatal accident and cause-3 $\left(FA_{-1}=>C3_{-1}=>FA_{-1}=FA_{-1}\Leftrightarrow C3_{-1}\right)$ with a difference of one at a 5% level of significance and unidirectional causality with a difference of three $\left(C3_{-3}=>FA_{-3}\neq C3_{-3}\right)$ is significant at the 1% level. With a difference of two and three, a unidirectional relationship between cause-1 and cause-2 is found $\left(C1_{-2,-3}=>C2_{-2,-3}\neq C1_{-2,-3}\right)$ at a 1% level of significance. The unidirectional relationship between cause-1 and cause-4 $\left(C1_{-2}=>C4_{-2}\neq C1_{-2}\right)$, cause-3 and cause 1$\left(C3_{-1}=>C1_{-1}\neq C3_{-1}\right)$, cause-3 and cause-2 $\left(C3_{-1,-2}=>C2_{-1,-2}\neq C3_{-1,-2}\right)$, cause-4 and fatal accidents $\left(C4_{-1,-2}=>FA_{-1,-2}\neq C4_{-1,-2}\right)$, cause-4 and cause-1 $\left(C4_{-1,-2,-3}=>C1_{-1,-2,-3}\neq C4_{-1,-2,-3}\right)$, cause-4 and cause-2$\left(C4_{-1,-2,-3}=>C2_{-1,-2,-3}\neq C4_{-1,-2,-3}\right)$, and cause-4 and cause-3 $\left(C4_{-1,-3}=>C3_{-1,-3}\neq C4_{-1,-3}\right)$ with different subscripts showing differences are declared by analysis and presented in Table 6. The speed of adjustment from short-term equilibrium to long-term equilibrium for fatal accidents is 42.60%, with a negative sign and a 10% significance level. Table 7 is a summary of Table 6 and cross-check of directional causality. The VECM declared the long-run relationship among variables and the speed of variables’ adjustment from the short-run to the long-run equilibrium level. The unidirectional relationship is shown with “→” and the opposite or bidirectional relationship with “~” in Table 7. The total observations of the research are 37.

Study results show a relationship between work accidents, causes dependent on the executor, causes dependent on the means of production, and work-dependent causes with 1% and 5% significance levels. In fatal accidents, cause-1 (dependent on the executor) and cause-3 (workload-dependent causes) have shown a positive and significant relationship with a fatal accident at a 5% level. Cause-2 (dependent on the means of production) and cause-4 (work-dependent causes) have shown a positive and insignificant relationship with fatal accidents.

**Figure 3:**
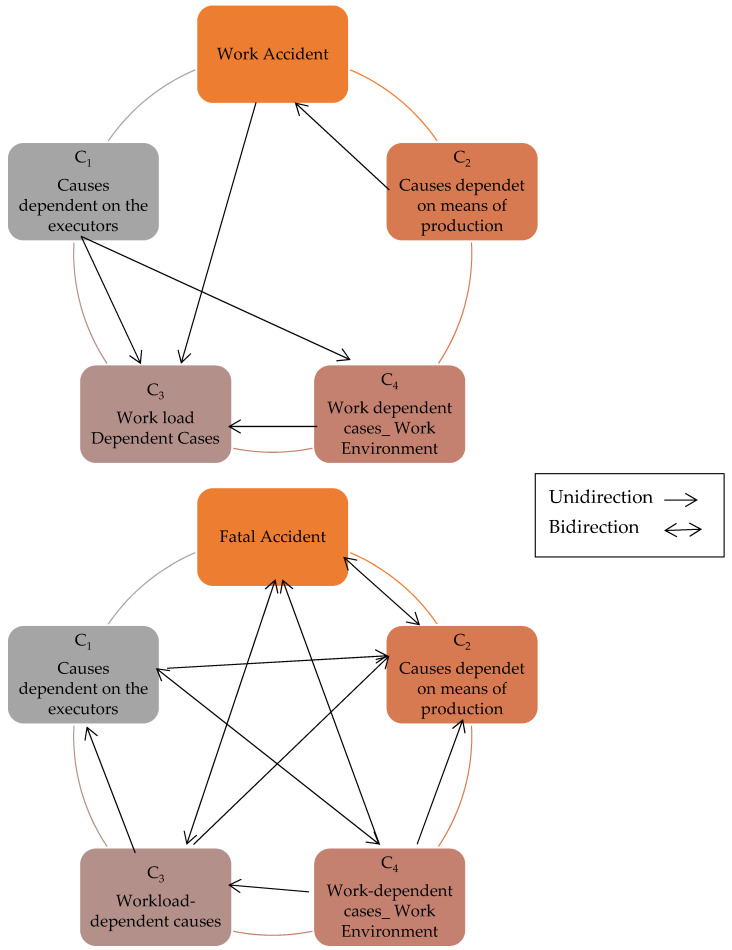
Summary of Granger causality tests for short run.

Figure 3 is a graphical representation of the short-run relationship between work accidents and fatal accidents with selected independent causes, i.e., the injured person (executor), means of production, workload, and work environment.

## 4. Discussion

Organizational activities contain many hazardous steps collectively, which are known as organizational risk. Improper management of organizational risk can decrease an organization’s production, affecting the speed of the production process and work accidents [48]. This research is focused explicitly on accidents (fatal and non-fatal) as dependent variables, while different causes of accidents are considered as independent variables [49,50,51]. Working equipment is also vital in workplace accidents [52]. Many causes will become the reason for accidents in an organization, but our selected causes are related to executors, means of production, workload, and work dependent on the work environment. Analytical data were collected from the Romanian firms. First of all, the ADF is used to check the stationarity of the data series. The stationarity of both series under work and the fatal accident is confirmed after taking the first difference. Both conditional series have shown their significance at a 1% level for all causes except C-2 of the fatal accident. The C-2 of the fatal accident is significant at a 5% level of confidence. Secondly, the appropriate lag length is selected for both time series by considering lag length criterions, i.e., Akaike information criterion (AIC), Schwarz information criterion (SC), and the Hannan–Quinn information criterion (HQ). For work accidents, all criterions support lag three as an appropriate lag length.

In contrast, fatal accidents receive support for lag three by two criterions (AIC and HQ), and SIC chose lag 0 as an appropriate lag. Thirdly, the Johansen cointegration test is employed to check the long-run relationship among variables. The Johansen cointegration confirmed a long-run integration among data series. Lastly, the vector error correction model (VECM) was applied to measure the speed of adjustment from short run to long run, which is higher for work accidents (83.35%) than fatal accidents (42.60%). As per our analysis, work accidents are riskier and more impactful than fatal accidents, and a close and strong relationship between work accidents and their causes is observed. Heinrich’s law has also supported the results of this research as Heinrich’s law categorized 0.3% accidents as having majorly injured the victim, 8.8% having been minorly injured, and 90.9% having no injuries. According to this pre-mentioned rule, the ratio between work accidents and injuries and fatal accidents and injuries comparatively increases at the same rate [53]. The short-run causality test shows unidirectional causality between work accident and cause-3 with a first difference $\left(WA_{-1}=>C3_{-1}\neq WA_{-1}\right)$ at a 10% level of significance. The unidirectional causal effect of cause-1 and cause-2 on work accidents $\left(C2_{-1}=>WA_{-1}\neq C2_{-1})\;(C1_{-3}=>WA_{-3}\neq C1_{-3}\right)$ with different subscripts is confirmed at a 1% level of significance. The unidirectional relationship observed between fatal accident and cause-2 with one difference $\left(FA_{-1}=>C2_{-1}\neq FA_{-1}\right)$ at 1% and cause-2 with a difference of three $\left(C2_{-3}=>FA_{-3}\neq C2_{-3}\right)$ is significant at a 5% level of significance. A bidirectional relationship observed between fatal accident and cause-3 $\left(FA_{-1}=>C3_{-1}=>FA_{-1}=FA_{-1}\Leftrightarrow C3_{-1}\right)$ with a difference of one at a 5% level of significance and unidirectional causality with a difference of three $\left(C3_{-3}=>FA_{-3}\neq C3_{-3}\right)$ is significant at the 1% level. The unidirectional relationship between cause-4 and fatal accidents $\left(C4_{-1,-2}=>FA_{-1,-2}\neq C4_{-1,-2}\right)$ is observed.

### Study Limitations and Future Research Directions

As per our best knowledge, this is the first research work that pointed out the relationship direction between work and fatal accidents and their causes by utilizing the data of Romanian organizations. Due to the innovative nature of research and direction, this research is limited in its context. Firstly, the study is focused only on Romanian industries as a whole, while some productive activities are riskier in different organizations than others. In microanalysis, future research can be focused on accidental risk in the different industries separately. The microanalysis will determine the exact practical place or activity that shows accidental risk management’s quick impact. Secondly, the macro impact of this research can be checked in future research by enhancing the data series, focused on all types of industries, including manufacturing industries, comparative analysis of manufacturing industries and rest of all industries, and comparative analysis of different Romanian industries with other developed countries. Thirdly, the ARDL methodological approach can analyze future prediction capability and check the psychological impact of work accidents on industries employees using the sentiment index approach. Future research will address the costs of occupational and fatal risks. This research employed the Johansen cointegration model, vector error correction, and Granger causality models. The GMM, panel regression analysis, and dynamic ARDL models can be employed concerning work accidents in future research. The same study can be done with various databases from different countries and compare them.

## 5. Conclusions

Accidents at work are of great importance to organizations. These must be addressed through risk management to be planned, evaluated, analyzed, and controlled correctly. Regardless of the field of activity, human resources are exposed to the causes that contribute to risks. The treatment of the causes contributes to reducing the probability of occurrence of the risks and implicitly to the diminution of the consequences. Once a risk has arisen, the foreseeable consequences must be considered. In this research, the augmented Dickey–Fuller (ADF) test is employed to check data series stationarity. This research demonstrated that both data series are free from the unit-root problem at first difference. The lag length criterions select the third lag for model fitness, and Johansen cointegration declares that variables are cointegrated for the long term.

The short-run directional nature of variables shows unidirectional causality between work accidents and workload-dependent causes, with the first difference at a 10% significance level. The unidirectional relationship observed between fatal accident and the injured person with one difference at 1% and means of production with a difference of three significant at a 5% level of confidence. A bidirectional relationship was observed between fatal accidents and workload-dependent causes with a difference of one at a 5% level of significance. A unidirectional causality of workload toward fatal accidents with a difference of three is confirmed. The unidirectional relationship between work environment and fatal accidents is observed. The VECM declares the speed of adjustment from the short to the long run is 83.35% and 42.60% for work and fatal accidents. Future research will address the costs of occupational and fatal risks.

## Figures and Tables

**Table 1:** Data description.

Main Causes	Work Conditions
**Cause-1** Causes dependent on the executor—the injured persons (the executors)	Falls
	Improper performance of work operations
	Omissions (failure to use the means of protection provided; failure to perform some operations essential to occupational safety promptly)
	Exposure, outside the workload, too dangerous or harmful factors (travel, parking in places or areas with temporary or permanent danger)
	Carrying out, outside the workload, operations that result in dangerous or harmful conditions
	Presence at work in inappropriate psycho-physiological conditions
	Other causes
**Cause-2** Causes dependent on the means of production	Physical causes (movements under the effect of gravity; functional movements of machines and mechanisms, etc.)
	Chemical causes (danger of contact or handling)
	Causes of nature biological (danger of contact or handling)
**Cause-3** Workload-dependent causes	Deficiencies in guidance, supervision, and control
	Errors in the predetermination of work operations
	Omissions in the predetermination of labor operations
	Deficiencies in ensuring the conditions of occupational safety and health
	Improper distribution of performers in the workplace
	Other causes
**Cause-4** Work-dependent causes—the work environment	Physical causes (air temperature, air humidity, low light level)
	Psychosocial climate
	The special character of the environment
	Other causes

**Table 2:** Augmented Dickey–Fuller test.

**Stationarity Test of Work Accident Causes**						**Order of** **Cointegration**
**Variables**	**Unit-Root Test**	**Augmented Dickey–Fuller Test (Intercept)**		**ADF (Trend and Intercept)**		**ADF**
		**ADF t-Stat**	***p*-Value**	**ADF t-Stat**	***p*-Value**	
**WA**	Level Data	−0.3811	0.9019	−3.0284	0.1400	I(1)
	1st difference data	−11.1113 *	0.0000	−11.0411 *	0.0000	
**C-1**	Level Data	−0.6419	0.8485	−1.4669	0.8225	I(1)
	1st difference data	−50.1946 *	0.0001	−49.2384 *	0.0000	
**C-2**	Level Data	−0.2699	0.9195	−2.1816	0.4845	I(1)
	1st difference data	−21.1863 *	0.0001	−20.9114 *	0.0000	
**C-3**	Level Data	−1.7557	0.3956	−2.6124	0.2774	I(1)
	1st difference data	−10.9289 *	0.0000	−10.7576 *	0.0000	
**C-4**	Level Data	−2.3014	0.1770	−2.3132	0.4165	I(1)
	1st difference data	−13.1573 *	0.0000	−12.9533 *	0.0000	
**Stationarity Test of Fatal Accident Causes**						
**FA**	Level Data	−2.1382	0.2316	−2.5677	0.2963	I(1)
	1st difference data	−17.5672 *	0.0000	−20.7954 *	0.0000	
**C-1**	Level Data	−2.0521	0.2644	−1.9677	0.5981	I(1)
	1st difference data	−7.8011 *	0.0000	−7.6647 *	0.0000	
**C-2**	Level Data	−0.0050	0.9508	−3.1082	0.1220	I(1)
	1st difference data	−3.0923 **	0.0379	−2.2802 **	0.0241	
**C-3**	Level Data	−1.4592	0.5424	−1.2104	0.8932	I(1)
	1st difference data	−8.3599 *	0.0000	−8.3354 *	0.0000	
**C-4**	Level Data	0.1470	0.9646	−2.0037	0.5796	I(1)
	1st difference data	−17.3718 *	0.0001	−17.2347 *	0.0000	

* and ** are representative of a 1% and 5% level of significance, respectively. C represents the main causes, WA is a work accident, and FA is a fatal accident.

**Table 3:** VAR lag order selection criteria.

**Work Accident**						
**Lag**	**Log L**	**LR**	**FPE**	**AIC**	**SC**	**HQ**
**0**	−46.4875	NA	1.11 × 10^−^^5^	2.7831	3.0008	2.8598
**1**	1.4892	80.3934	3.26 × 10^−^^6^	1.5411	2.8472	2.0016
**2**	72.1194	99.2641	2.99 × 10^−^^7^	−0.9253	1.4692	−0.0811
**3**	117.3881	51.3860 *	1.24 × 10^−7^ *	−2.0209 *	1.4620 *	−0.7930 *
**Fatal Accident**						
**0**	−637.086	NA	8.14 × 10^8^	34.7073	34.9250 *	34.7840
**1**	−619.373	29.6798	1.23 × 10^9^	35.1012	36.4074	35.5617
**2**	−582.281	52.1303	6.89 × 10^8^	34.4476	36.8422	35.2918
**3**	−517.67	73.3417 *	1.00 × 10^8^ *	32.3064 *	35.7895	33.5344 *

* is a representative of a 1 level of significance.

**Table 4:** (**A**): Unrestricted cointegration rank test (trace). (**B**): Unrestricted cointegration rank test (maximum eigenvalue).

(**A**)			
**Work Accident**			
**Hypothesized No. of CE(s)**	**Trace Statistic**	**Critical Value (0.05)**	**Prob**.
None	83.83758 *	69.81889	0.0025
At most 1	42.0070	47.8561	0.1585
At most 2	22.3922	29.7970	0.2772
At most 3	9.1974	15.4947	0.3474
At most 4	0.6467	3.8414	0.4213
**Fatal Accident**			
None	76.77265 *	69.81889	0.0125
At most 1	40.7012	47.8561	0.1984
At most 2	20.3516	29.7970	0.3993
At most 3	6.3039	15.4947	0.6596
At most 4	0.1497	3.8414	0.6988
(**B**)			
**Work Accident.**			
**Hypothesized No. of CE(s)**	**Max Eigen Statistic**	**Critical Value (0.05)**	**Prob.**
None	41.8304 *	33.87687	0.0046
At most 1	19.6148	27.5843	0.3684
At most 2	13.1947	21.1316	0.4345
At most 3	8.5506	14.2646	0.3254
At most 4	0.6467	3.8414	0.4213
**Fatal Accident**			
None	36.0714 **	33.87687	0.0269
At most 1	20.3495	27.5843	0.3175
At most 2	14.0477	21.1316	0.3611
At most 3	6.1542	14.2646	0.5935
At most 4	0.1497	3.8414	0.6988

* and ** are representative of a 1%, 5%, and 10% level of significance, respectively.

**Table 5:** Normalized cointegration coefficient.

**Work Accident**				
**Cointegration Equation(s)**	**C-1**	**C-2**	**C-3**	**C-4**
**1.000**	−1.9710	−3.2879	0.9780	1.2224
**Standard Error**	(−1.5251)	(−0.7474)	(−0.3678)	(−0.2899)
Log-likelihood			146.8347	
**Fatal Accident**				
**1.000**	−1.9711	−3.2879	0.9780	1.2224
**Standard Error**	(−1.5251)	(−0.7474)	(−0.3678)	(−0.2899)
Log-likelihood			−459.1769	

C represents the main causes, WA is a work accident, and FA is a fatal accident.

**Table 6:** Vector error correction estimates test.

**The Direction of Causality for Work Accident**						
	**Short Run**					**Long Run**
**Error Correction**	**WA/FA**	**C-1**	**C-2**	**C-3**	**C-4**	**ECT_t−1_**
D(WA (-1))		0.0066	−0.3599	1.0145 ***	0.3901	-0.8335 *
D(WA (-2))	------	0.0267	−0.3134	0.2926	0.4813	
D(WA (-3))		0.0070	−0.2272	−0.1102	0.3494	
D(W_ C-1(-1))	0.3167		0.0269	−1.8422 *	−0.422 ***	0.8393 *
D(W_ C-1(-2))	0.1325	-------	0.3145	−2.1211 *	−0.0255	
D(W_ C-1(-3))	0.7307 *		−0.0308	−1.9391 *	−0.0358	
D(W_ C-2(-1))	−0.3793 *	−0.0314		0.1708	−0.0286	0.4489 ***
D(W_ C-2(-2))	−0.0972	−0.0560	-------	0.1891	−0.0677	
D(W_ C-2(-3))	0.0421	−0.0720		0.0230	−0.0474	
D(W_ C-3(-1))	−0.0455	0.0008	−0.1912		0.2565	-0.2808
D(W_ C-3(-2))	−0.0716	0.0385	−0.1366	-------	0.1598	
D(W_ C-3(-3))	−0.0888	−0.0051	0.0328		0.0525	
D(W_ C-4(-1))	0.2447	0.0426	0.4542	−0.0907 ***		0.6356 *
D(W_ C-4(-2))	0.2169	0.0447	−0.1183	0.0640 **	------	
D(W_ C-4(-3))	0.1469	0.0093	−0.2053	0.6393 ***		
C	0.0242	0.0557 *	0.0647 ***	0.0443	−0.0160	-----
**The Direction of Causality for Fatal Accident**						
D(FA (-1))		−0.0429	−0.2644 *	0.2921 **	−0.1134	−0.4260 ***
D(FA (-2))	------	0.0159	-0.0430	0.1511	0.0567	
D(FA (-3))		−0.0018	−0.0364	0.0778	0.2439	
D(F_ C-1(-1))	−1.8070		1.8087	−0.0352	−0.6500	0.0463
D(F_ C-1(-2))	−0.2252	------	2.9347 *	−1.2752	3.0441 **	
D(F_ C-1(-3))	−0.6185		1.5921 *	−1.5642	1.9980	
D(F_ C-2(-1))	−1.2928	0.1225		−0.7592	−0.3945	0.3532 *
D(F_ C-2(-2))	−0.6686	0.0195	------	0.0948	−0.1003	
D(F_ C-2(-3))	1.3170 **	0.0551		−0.8216 **	−0.3172	
D(F_ C-3(-1))	−1.161 **	−0.1242 ***	−0.4859 *		−0.2426	−0.1377
D(F_ C-3(-2))	−0.4307	−0.0436	−0.2981 ***	------	−0.4015	
D(F_ C-3(-3))	1.2457 *	−0.0504	−0.0359		0.3588	
D(F_ C-4(-1))	0.6982 **	−0.1336 *	−0.6743 *	0.4197 ***		0.1143
D(F_ C-4(-2))	0.5111 **	−0.0851 ***	−0.2427 *	0.0266	------	
D(F_ C-4(-3))	−0.0330	−0.0905 ***	−0.4122 *	0.3943 *		
C	0.6565	0.2089	0.0104	0.4052	2.7403	----

*, **, and *** are representative of a 1%, 5%, and 10% level of significance, respectively. C represents the main causes, WA is a work accident, and FA is a fatal accident.

**Table 7:** Granger causality test between selected causes.

**Work Accident**					
**Direction of Causality**			**Observations**	**F-Statistics**	**Prob.**
C-1	→	WA	37	10.8294 *	6.00 × 10^−^^5^
WA	→	C-1		8.9783 *	0.0002
C-2	→	WA	37	12.5607 *	2.00 × 10^−^^5^
WA	→	C-2		5.6678 *	0.0034
C-3	→	WA	37	4.1349 *	0.0144
WA	~	C-3		1.5978	0.2106
C-4	→	WA	37	5.6942 *	0.0033
WA	→	C-4		3.3517 **	0.0319
**Fatal Accident**					
C-1	→	FA	37	3.5962 **	0.0248
FA	~	C-1		1.1920	0.3295
C-2	~	FA	37	0.7898	0.5091
FA	~	C-2		1.7937	0.1696
C-3	~	FA	37	1.8127	0.1661
FA	→	C-3		4.0062 **	0.0164
C-4	~	FA	37	2.1459	0.1152
FA	~	C-4		1.5999	0.2101

## Data Availability

The data of current study can be obtained from corresponding author.
